# Two-Hit Kidney Allograft Injury by SARS-CoV-2

**DOI:** 10.7759/cureus.34603

**Published:** 2023-02-03

**Authors:** Bárbara Ribeiro, Marina Pontello Cristelli, Renato Demarchi Foresto, Henrique Machado Proença, José Medina-Pestana

**Affiliations:** 1 Nephrology Department, Hospital de Braga, Braga, PRT; 2 Nephrology and Kidney Transplantation Department, Hospital do Rim, Fundação Oswaldo Ramos, São Paulo, BRA

**Keywords:** renal allograft dysfunction, collapsing glomerulopathy, acute thrombotic microangiopathy, kidney transplantation, covid-19

## Abstract

Coronavirus disease 2019 (COVID-19) has been associated with acute kidney injury in kidney transplant recipients by several mechanisms. The authors report a case of acute kidney allograft dysfunction in a 48-year-old patient who presented in the emergency room with anasarca and nephrotic syndrome close after mild COVID-19 and no other clinical condition. Histopathology of the allograft biopsy revealed two distinct and simultaneous kidney lesions, collapsing glomerulopathy and thrombotic microangiopathy. Renal function persistently deteriorated, and definitive dialysis was initiated. After excluding other plausible causes for the findings, this case strengthens the hypothesis that the kidney allograft is also a target of severe acute respiratory syndrome coronavirus 2 (SARS-CoV-2).

## Introduction

In the general population, severe coronavirus disease 2019 (COVID-19) has been frequently associated with acute kidney injury (AKI), mostly due to the mechanisms of lesions associated with hypotension, sepsis, and/or cytokine storm in the context of the disease. However, other clinicopathological pictures have been described, such as collapsing glomerulopathy and thrombotic microangiopathy [1-4]. Interestingly, these lesion patterns can occur even in the context of mild disease, suggesting that the human kidney is a specific target for severe acute respiratory syndrome coronavirus 2 (SARS-CoV-2) infection. Supporting this hypothesis, previous findings show that the receptors used by the virus to enter the cells, namely angiotensin conversing enzyme-2 (ACE2) and CD147, are highly expressed in the podocytes and epithelial tubular cells [5]. Furthermore, clusters of viral particles were also found in these types of kidney cells by electron microscopy in autopsy series, and *in situ* hybridization and immunohistochemistry techniques prove that the virus accumulates in the tubules [6]. Among kidney transplant recipients, different forms of renal involvement have also been observed, with variable impacts on long-term kidney function, such as acute rejection, collapsing glomerulopathy, and lupus podocytopathy [1-3,7-10]. These cases presented mainly with acute allograft dysfunction and proteinuria. Herein, we report a case of a transplant recipient who developed severe nephrotic syndrome in the context of oligosymptomatic COVID-19, leading to allograft loss and drawing attention to the coexistence of two distinct histopathological lesions.

## Case presentation

A 48-year-old Caucasian woman was admitted to the emergency room with progressive generalized edema, anorexia, and asthenia for seven days. She also reported headache, runny nose, and dyspnoea 15 days before hospital admission. She had a medical history of end-stage renal disease (ESRD) of unknown cause and had received a deceased-donor kidney transplant eight months ago. She had completed a three-dose schedule of coronavirus vaccine (CoronaVac®, Sinovac Life Sciences, Beijing, China) four months earlier and had no other relevant past medical history. The patient was under immunosuppression with tacrolimus, mycophenolate mofetil, and prednisone. Her baseline serum creatinine was 1.7 mg/dL (estimated glomerular filtration rate by Chronic Kidney Disease Epidemiology Collaboration [CKD-EPI] formula of 35 mL/min/1.73m²). At admission, physical examination was unremarkable other than anasarca, with peripheral oxygen saturation of 95% and clear pulmonary auscultation. Blood analysis revealed white blood cells count (WBC) of 10.7 × 10⁹/L, hemoglobin of 10.3 g/dl, platelets of 247,000/m³ (reference range [RR]: 150,000-450,000/m³), C-reactive protein (CRP) of 0.51 mg/dL (RR: 0-0.11 mg/dL), lactate dehydrogenase (LDH) of 260 UI/L (RR: 140-270 UI/L), creatinine (Cr) of 6.3 mg/dL, albumin of 2.9 g/dl, tacrolimus trough blood concentration of 8 ng/mL, C3 of 83 mg/dl (RR: 87-200 mg/dl), C4 of 32 mg/dl (RR: 19-52 mg/dl). The urinary protein-creatinine ratio was 15.6. Polymerase chain reaction (PCR) assay for SARS-CoV-2 from nasopharyngeal swab resulted positive (cycle threshold of 24.18). Chest computed tomography (CT) showed scarce small nodules, some with a ground-glass halo, and other focal opacities, some ground-glass, also scattered throughout the lung lobes, predominantly in the right upper lobe.

**Figure 1:**
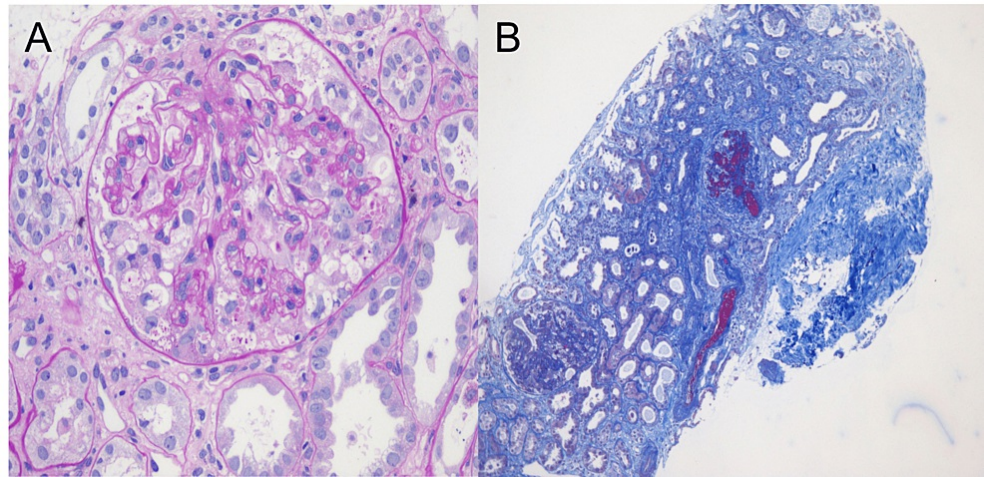
Kidney allograft biopsy findings (A) Light microscopy showing a lesion of collapsing glomerulopathy characterized by hyperplasia of glomerular epithelial cells and wrinkling of the glomerular basement membranes resulting in the collapse of the glomerular tuft (periodic acid-Schiff stain, x400). (B) Light microscopy also demonstrates multiple microthrombi into the glomerular capillary loops and extensive fibrin thrombi occluding the lumen of an arteriolar vessel (Masson trichrome stain, x100).

A percutaneous kidney biopsy was performed two days after admission revealing collapsing glomerulopathy in two of the nine glomeruli samples with the global collapse of the capillary tuft accompanied by pronounced podocyte hyperplasia and hypertrophy and swelling of epithelial cells (Figure 1). Additionally, the sample revealed in another two glomeruli the presence of arteriolar and glomerular capillary thrombi with signs of mesangiolysis, which was consistent with acute microangiopathy (Figure 1). Interstitial fibrosis and tubular atrophy were present in 60% of the sample (Figure 1). Immunofluorescence assay of glomeruli did not reveal immune deposits, including C4d, and electron microscopy was not performed. Donor-specific anti-human leukocyte antigens (HLA) antibodies were absent. Serologic tests for human immunodeficiency virus (HIV), hepatitis B and C viruses were negative, and PCR testing of blood samples for cytomegalovirus, Epstein-Barr virus, parvovirus B19, and BK polyomavirus were undetectable. Genetic analysis of apolipoprotein L-1 (APOL1) was unavailable.

From the respiratory point of view, the patient had a benign evolution, with no need for oxygen therapy during her hospitalization. However, facing the severity of kidney dysfunction and the histological findings, supportive treatment was instituted with dialysis, volemia control, and antihypertensive treatment. No specific measure was applied, and the patient maintained surveillance for infectious and late COVID-19 complications. After 21 days of hospitalization, the patient was discharged, after resolution of edema, constitutional and respiratory symptoms, under chronic hemodialysis and was reincluded in the kidney transplant waiting list. 

## Discussion

In the general population, it is postulated that SARS-CoV-2 can cause kidney injury by diverse mechanisms, including interaction with its cellular receptor ACE-2, virus-induced systemic cytokine storm, prothrombotic effects and direct viral invasion [1-3,11,12]. The hypothesis that the kidney might be a target of the SARS-CoV-2 virus is supported by the fact that ACE-2 is abundantly present in kidney tissue, mostly in podocytes and in the brush border of the proximal tubule, the isolation of the virus from the urine of infected patients, and the findings of particles resembling coronaviruses in proximal tubule cells, by transmission electron microscopy [13-15].

Among kidney transplant recipients, COVID-19 has been demonstrated to directly result in high morbidity and mortality, with an attributed fatality rate ranging from 20% to 30% in the pre-omicron era [16-18]. There is a paucity of information about COVID-19-associated pathology in kidney transplant patients, and different forms of renal involvement can be histologically detected after allograft biopsy [7-10]. COVID-19-associated collapsing glomerulopathy is a predominant finding in the limited published literature and has been linked to African descent patients with high-risk APOL1 alleles [1,2]. Microthrombi in glomerular tufts and arterioles were also observed in kidney transplant biopsies in the setting of COVID-19 and may be a consequence of a hypercoagulability that accompanies the infectious disease [8,19,20]. Histologic evidence of acute rejection has also been reported, especially acute vascular rejection with higher Banff scores for arteritis and glomerulitis [2-3].

In the present report, the kidney allograft biopsy revealed coexistent collapsing glomerulopathy and thrombotic microangiopathy (TMA). In a series of 17 kidney biopsies (14 of native kidney and three of transplant kidney) performed in COVID-19 patients from seven large centers in the United States, two of the native kidney biopsies also revealed both lesions, whose patients presented with nephrotic syndrome and severe AKI, progressing to kidney failure and dialysis [3]. In another series of 18 kidney allograft biopsies performed in the setting of COVID-19 at Columbia University, one revealed collapsing glomerulopathy and showed TMA, with glomerulus collapse being interpreted as secondary to acute microangiopathy [2].

It is possible that the podocytopathy and microcirculation thrombotic lesions resulted from two distinct mechanisms, as the lesions appeared in different glomeruli. On the one hand, the virus may have promoted direct or indirect podocyte injury through ACE-2 receptor expressed by podocytes, cytokine-mediated injury, or enhanced type 1 interferon expression, this last resembling HIV nephropathy. On the other hand, the presence of microthrombi in glomerular tufts and mesangiolysis indicated endothelial injury, possibly from direct viral toxicity to the endothelium or through uncontrolled activation of the alternative complement pathway by the infection, acting as a second hit, unmasking an underlying genetic defect, which unfortunately was not evaluated in this case.

## Conclusions

The authors describe a clinical case where two distinct lesions, rarely observed in kidney transplant recipients with mild COVID-19, occurred simultaneously with a poor allograft outcome. Although causality between viral infection and subsequent glomerulopathy cannot be proven, this report warns that not all renal dysfunctions following infection are due solely to tubular compartment injury. In addition, it reflects that unfavorable transplant outcomes are possible even in a mild COVID-19 setting.
